# Maternal Vitamin D Deficiency and the Risk of Small for Gestational Age: A Meta-analysis

**Published:** 2018-12

**Authors:** Zhao HU, Lu TANG, Hui-Lan XU

**Affiliations:** Dept. of Social Medicine and Health Management, Xiangya School of Public Health, Central South University, Changsha 410078, China

**Keywords:** Vitamin D, Small for gestational age, Pregnancy

## Abstract

**Background:**

Inconsistencies among studies still exist in regard to the associations between maternal vitamin D deficiency and the risk of small for gestational age.

**Methods:**

We conducted a meta-analysis of observational studies to evaluate these associations. We searched electronic databases for literature published in PubMed, Medline, Web of Science, Embase and the Cochrane Library up to Dec 2016 using the following keywords: “vitamin D” or “cholecalciferol” or “25-OHD” or “25-hydroxyvitamin D” in combination with “small for gestational age” or “SGA” or “fetal outcome” or “pregnancy outcome”.

**Results:**

Twelve studies involving 19,027 participants were included in this meta-analysis. Pregnant women with vitamin D deficiency (25-OHD levels<50 nmol/L) experienced an increased risk of SGA (odds ratio (OR) =1.41, 95% confidence interval (CI): 1.14, 1.75). The vitamin D concentration of the SGA group was lower than that of the non-SGA group (mean difference: −1.75 nmol/L, 95%CI: −3.23, −0.27).

**Conclusion:**

Maternal vitamin D deficiency during pregnancy may be associated with an increased risk of SGA.

## Introduction

Vitamin D deficiency is a common and increasing problem all over the world, and concerns have been raised that there may be an epidemic of such deficiency in pregnancy (1, 2). Poor vitamin D status during pregnancy was associated with an increased risk of many adverse obstetric outcomes, including insulin resistance (3), gestational diabetes mellitus (4, 5), preeclampsia (6), bacterial vaginosis (7), preterm delivery (8), and cesarean section (9). Moreover, deficient vitamin D status during pregnancy may have adverse consequences for offspring development and growth explored (10, 11).

Small for gestational age (SGA) is usually defined as neonates with birth weights below the 10^th^ percentile for their gestational age or/and length at least 2 standard deviations below the mean for gestational age (12). Small for gestational age increased the risk of infant morbidity and metabolic disease in adulthood (13, 14). However, the link between vitamin D status and small for gestational age has been explored by a number of studies worldwide with mixed results. Vitamin D deficiency during pregnancy is associated with an increased risk of small for gestational age. For instance, pregnant women with vitamin D concentrations less than 20 ng/mL had 6.47 times (95%CI: 4.30, 9.75) higher risk of SGA compared to those with vitamin D concentrations greater than 30 ng/mL (15). Women with deficient vitamin D levels had infants with a higher risk of SGA (OR=2.4, 95%CI:1.9,3.2) (16). In contrast, other studies (17–19) demonstrated no significant associations between vitamin D deficiency and SGA. Thus, a meta-analysis showed that insufficient serum levels of 25-OHD were associated with several adverse pregnancy outcomes including SGA (20). However, this study included studies published before year 2012 and not analyzed the mean difference of 25-OHD between SGA and non-SGA group. In addition, they did not analyze the associations between different cut-offs of vitamin D deficiency and risk of SGA. Furthermore, it also not conducted sensitivity analyses to explore potential sources of heterogeneity.

Therefore, we carried out this meta-analysis aim to evaluate the association between vitamin D deficiency and risk of SGA though providing more precise estimation and more sufficient evidence.

## Methods

This meta-analysis was conducted and reported in accordance with the STROBE checklist (Strengthening the Reporting of Observational Studies in Epidemiology) (21).

### Data sources

We performed a systematic literature search of PubMed, Web of Science, Medline, Embase and the Cochrane Library using the following search limiters: publication studies from Jan 2006 to Dec 2016, studies in humans and studies written in English. To search for the literature between vitamin D and risk of SGA comprehensively, we used the following search syntax: (“vitamin D” or “cholecalciferol” or “25-hydroxyvitamin D” or “25-OHD”) and (“small for gestational age” or “SGA” or “fetal outcome” or “pregnancy outcome”).

Additionally, we checked the reference lists of identified reports for other potentially relevant studies. We also contacted the authors to ask for additional information and unpublished data as needed.

### Eligibility criteria and study selection

Original articles exploring the relationship between maternal vitamin D and pregnancy outcomes were reviewed and included if they met the following criteria: (a) the study population included individuals up to 16 yr of age; (b) subjects were pregnant women without HIV infection, syphilis infection or severe illness; (c) women with singleton gestation were included; (d) maternal blood samples were taken for assays of 25 (OH)D during pregnancy or at delivery; (e) small for gestational age was the pregnancy outcome, and the control group consisted of women without SGA; (f) SGA was defined as neonates with birth weights below the 10^th^ percentile for their gestational age but not below the 5^th^ percentile for gestational age; (g) vitamin D deficiency was defined as a 25-OHD level below 50 nmol/L (20ng/mL); (h) the subjects were pregnant women without dietary or exercise intervention during pregnancy; (i) sample size, SGA events and odd ratios (OR) or relative risk (RR) with 95% confidence intervals (CI) were presented, or information was provided used to infer these results.

### Data extraction and study quality evaluation

Two reviewers screened abstracts and titles to identify whether articles needed further review. To determine which papers were to be included, two reviewers independently screened the full text of identified articles against the inclusion criteria. An article was retained if either reviewer believed that it should be retained. We resolved any disagreements through consensus or arbitration by a third reviewer (XHL). We developed a data extraction procedure from included studies to collect key indicators of study quality using meta-analysis of observational studies in epidemiology standards. We extracted the characteristics of each included study, including the first author’s last name, year of publication, study design, location of study, sample size, events of SGA, methods of maternal 25-OHD assay, the level of vitamin D between SGA and non-SGA groups, prevalence of vitamin D deficiency, gestational age of sampling and the confounding factors included in the adjustments.

To assess the methodological quality of studies with an observational design, and in view of the fact that all but one of the studies reviewed had this type of design, we used the STROBE statement checklist to assess the methodological quality of the studies. Of the 22 items that make up the list, the 9 related to the methods section were selected (Appendix A). The methodological quality was classified as follows: articles that met 0–3 of 9 items were considered to have low methodological quality, those with 4–6 items had medium quality, and those with 7–9 items had high methodological quality.

### Statistical analysis

RevMan Software (version5.3, Cochrane Collaboration, London, UK) was used to generate pooled effects estimated for all outcomes with data from more than one eligible study. The association between vitamin D deficiency and SGA was reported in various ways, including proportions, odds ratios (95% confidence intervals (CI)), means (standard deviations), and median (inter-quartile ranges). Moreover, we converted medians and interquartile ranges into means and standard deviations using previously outlined methods if available (22). For studies with continuous outcomes, we generated mean differences, and for dichotomous outcomes, we calculated pooled odds ratios and 95%CI.

In studies that reported outcomes as proportions in two or more cut-off categories, we combined the numbers to create a category of deficiency for SGA, and we defined deficiency as a serum concentration less than 50nmol/L.

We used forest plots to visually assess pooled estimates effects and 95%CI for each study. Moreover, we quantified heterogeneity using *I*^2^ and the *I*^2^ statistic with a value >50% deemed to indicate substantial heterogeneity and *P<*0.05 considered statistically significant. Once the effect was found to be heterogeneous, a random effects model was used. Otherwise, a fixed effects model was used.

Funnel plots were used to examine the potential publication bias. Subgroup analysis and sensitivity analysis were performed to examine the possible reasons for heterogeneity. Subgroup analysis was conducted according to different study designs (cohort or nested case-control study) and gestational age of sampling. We also investigated the effect of a single article on the heterogeneity and overall risk estimated by removing one article in each turn. Two-tailed values of *P*<0.05 were considered statistically significant.

## Results

**Fig. 1:**
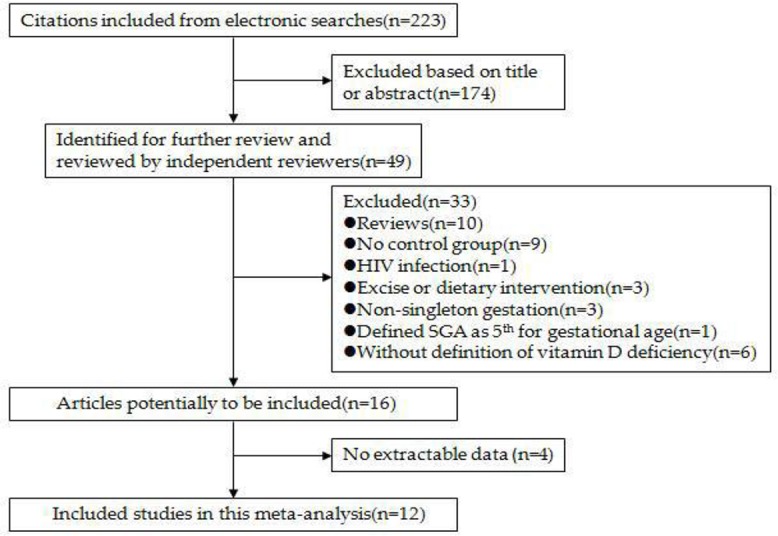
Flow of literature search and study selection

The literature search identified 223 articles pertaining to the association between maternal vitamin D and small for gestational age. After the initial screening of abstracts and titles, 49 papers were identified for further review by independent reviewers. After full-text review, 23 studies did not meet the inclusion criteria, 10 articles were reviews, and 4 articles had insufficient data. Finally, 12 studies were included in this meta-analysis. The screening process is shown in Fig. 1. The included studies meeting the STROBE checklist ranged from 7 to 9 items, which indicates that the methodological quality of included studies was medium or high. The results are shown in Table 1.

**Table 1:** Characteristics of observational studies included in this meta-analysis

***First Author (yr)***	***Country***	***Study Design***	***Sample Size***	***SGA (n)***	***Assay Method^1^***	***Mean 25-OHD(nmol/L)***		***GAS(weeks)^4^***	***Prevalence^5^***	***Adjustment^6^***	***MQ^7^***
						***SGA^2^***	***NSGA^3^***				
Bodnar (2010)(23)	USA	Nested case-control	1198	111	ELISA	NA^8^	NA	<22	NA	a,b,c,d,e,f,g,h,i	Medium
Burris (2012)(24)	USA	Prospective cohort	1303	53	CLIA,RIA	NA	NA	26–28	NA	a,c,d,j	Medium
Boyle (2016)(25)	New Zealand	Prospective cohort	1170	170	LC-MS	70.6±29.0	NA	15	21.5%	a,j	High
Fernandez-Alonso (2011)(26)	Spain	Prospective cohort	466	33	ECLIA	NA	NA	<14	23.4%	No	Medium
Gernand (2013)(27)	USA	Prospective cohort	2146	395	LC-MS	NA	NA	14–26	NA	a,b,c,d,f,j	Medium
Gernand (2014)(28)	USA	Prospective cohort	792	103	LC-MS	57.9±29.9	64.8±29.3	<26	NA	a,j,l	High
Leffelaar(2010)(16)	Netherlands	Prospective cohort	3730	343	EIA	NA	NA	12	21.4%	a,b,d,	High
Morgan(2016)(18)	Canada	Nested case-control	7929	301	CLIA	63.6±24.7	64.5±22.9	At delivery	NA	c,d	High
Ong(2016)(29)	Singapore	Prospective cohort	1247	120	LC-MS	NA	NA	26–28	13.2%	a,b,d,j	High
Schneuer(2014)(30)	Australia	Nested case-control	5109	388	AIA	55.3±20.0	56.9±19.9	<14	40.1%	b,c,d, m,n	High
Weinert(2016)(31)	Brazil	Prospective cohort	184	98	CLIA	NA	NA	26–28	53.3%	a,b,c	High
Zhou(2014)(17)	China	Prospective cohort	1953	11	ELISA	NA	NA	16–20	18.9%	No	Medium

1 Assay Method: AIA: automated immunoassay; CLIA: chemiluminescence immunossay; EIA: enzyme immunossay; ELISA: electrochemiluminescence immunossay; LC-MS: lipid chromatography-tandem mass spectrometry; RIA: radioimmunoassay; //

2 SGA: small for gestational age;

3 NSGA: non-small for gestational age; //

4 GAS: Gestational age of sampling;

5 Prevalence: prevalence of maternal vitamin D deficiency; //

6 Adjustments: a:pre-pregancy BMI; b: Smoking during pregnancy; c: season of blood draw; d: maternal age; e: gestational age at blood sampling; f: marital status; g: insurance status; h: smoking in the year before pregnancy; i: multivitamin use; j: race; k: gestational week of blood sample; l: allocation; m:materal weight; n:country of birth //

7 MQ: methodological quality;

8 NA: not available

### Study characteristics

The characteristics of the studies included in this meta-analysis are presented in Table 1. These studies were published from 2010 to 2016. Four studies were conducted in the USA, and one each in Spain, Singapore, Netherlands, New Zealand, Canada, China, Brazil, and Australia. Three articles were nested case-control study designs, and the rest were prospective cohort study designs.

According to the gestational age of sampling, three studies measured the maternal 25-OHD level in the first trimester (<14 wk). Five studies measured samples in the second trimester (14–26 wk), three studies measured samples in the third trimester (>26 wk) and one study collected samples at delivery.

Of these studies, ten explored the association between maternal vitamin D levels below 50 nmol/L and risk of SGA, and three studies explored the association between maternal vitamin D levels below 37.5 nmol/L and risk of SGA. However, only three studies explored the mean difference in maternal vitamin D levels between SGA and non-SGA groups.

Overall, data were available from 19027 participants, of whom 2218 (11.7%) were diagnosed with SGA. Additionally, the prevalence of vitamin D deficiency among pregnant women ranged from 13.2% to 53.3% if available.

### Main analysis

**Fig. 2:**
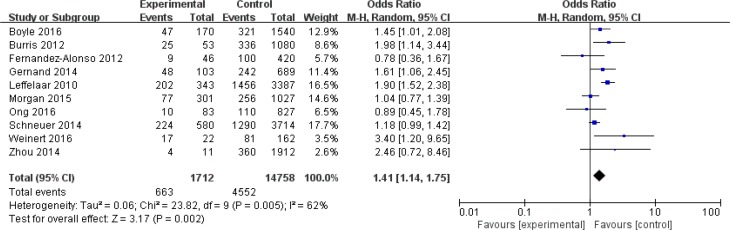
The association between maternal vitamin D deficiency and risk of SGA

The association between maternal vitamin D deficiency (25-OHD levels <50nmol/L) and risk of SGA is presented in Fig. 2.

Ten studies involving 16,470 pregnant women were included.

Small for gestational age was found to be associated with 25-OHD deficiency compared with the control group, with a pooled odds ratio based on a random effects model of 1.41 (95%CI:1.14,1.75) with moderate heterogeneity (*I*^2^=62%, *P*=0.005). Moreover, the pooled odds ratio was 1.30 (95%CI: 1.12, 1.52) between maternal vitamin D concentrations below 37.5 nmol/L and risk of SGA based on a fixed effects model (*I*^2^=0%, *P*=0.94).

**Fig. 3:**
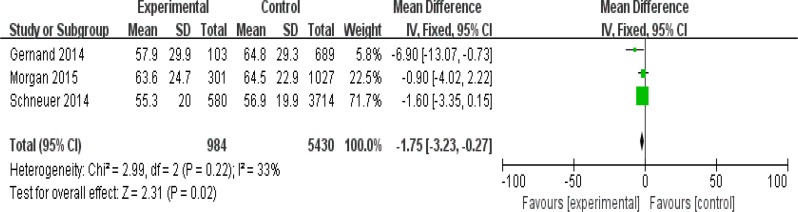
The association between maternal vitamin D level and SGA

In Fig. 3, we show a comparison of the mean difference between the SGA group (experimental group) and control group based on three studies involving 6414 pregnant women. The pooled effect was −1.75 nmol/L (95%CI: −3.23, −0.27) with low heterogeneity (*I*^2^=33%, *P*=0.22).

### Sensitivity and Subgroup Analysis

Heterogeneity was moderate in our meta-analyses (*I*^2^=62%, *P*=0.005). To explore the source of statistical heterogeneity of the included studies, sensitivity and subgroup analyses were conducted. A study was responsible for most of the heterogeneity (16). After excluding this study, heterogeneity was much lower among the remaining studies (*I*^2^=42%, *P*=0.09), and the pooled OR was 1.25 (95%CI:1.10, 1.41). Furthermore, there were no obvious changes in the pooled ORs as a result of the exclusion of any other single study. The pooled ORs ranged from 1.25(95%CI: 1.10, 1.41) to 1.49(95%CI: 1.19, 1.88), and each was statistically significant.

We conducted subgroup analysis between vitamin D deficiency and risk of SGA according to different study designs (cohort or nested case-control) and different gestational age of sampling (gestational age of sampling <16 or ≥16 wk). The pooled OR between vitamin D deficiency and risk of SGA was 1.14(95%CI: 0.98, 1.33) based on two nested case-control studies (*I*^2^=0%, *P*=0.45) and was 1.67(95%CI:1.44, 1.95) based on cohort studies (*I*^2^=39%, *P*=0.12). Additionally, the pooled OR between vitamin D deficiency and risk of SGA was 1.37(95%CI:1.00,1.88, when maternal blood was sampled to measure the levels of 25-OHD at gestational age <16 wk. The pooled OR was 1.49 (95%CI:1.05,2.12) at gestational age ≥ 16 wk.

We did not conduct a subgroup analysis of BMI, race, season, or location of study due to insufficient data in some studies.

### Bias

**Fig. 4:**
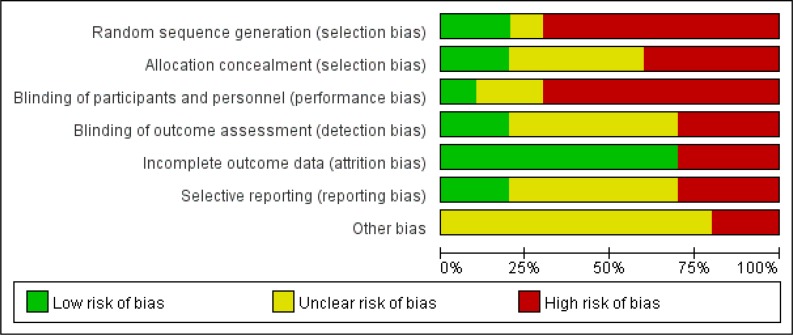
The risk of bias graph

Visual assessment of funnel plots showed that the studies were distributed fairly symmetrically about the combined effect size, showing that no obvious publication bias was observed in this meta-analysis. Additionally, the risk of bias graph is presented in Fig. 4.

## Discussion

Scientific understanding of the importance of maternal vitamin D during pregnancy for risk of SGA is growing rapidly, and our review consolidated a great deal of new information from observational studies not incorporated in a prior meta-analysis.

We performed a comprehensive search of PubMed, Medline, Web of Science, Embase and the Cochrane Library, which indexed the majority of scientific journals. Moreover, we searched these databases using a well-defined search strategy and we also screened the reference lists of all included studies and previous related reviews of this topic. For inclusion criteria of the studies, we formulated strict standards for subjects, a clear definition of SGA and a cut-off for vitamin D deficiency. We also detailed the methodological limitations of the included studies based on the STROBE checklist to ensure the quality of the evidence.

We systematically reviewed twelve observational studies that investigated the association between vitamin D deficiency and risk of SGA. From the limited evidence, maternal vitamin D deficiency (25-OHD below 50nmol/L) was associated with an increased risk of SGA(pooled OR=1.41, 95%CI:1.14,1.75), and the maternal vitamin D concentration of the SGA group was 1.75nmol/L less than the control group based on three studies. The findings of this pooled analysis are partially consistent with the positive association observed between vitamin D deficiency and risk of SGA in a meta-analysis conducted up to the year 2012 (32), which found that pregnant women with vitamin D concentrations less than 50 nmol/L experienced an increased risk of SGA (pooled OR=1.52,95%CI:1.08–2.15). Compared to that study, we covered the newest studies and conducted a subgroup analysis to explore potential sources of heterogeneity. Moreover, we also analyzed the mean difference of vitamin D levels between SGA and non-SGA groups.

In fact, the mean difference between the SGA and the non-SGA group was weak and may not be practically useful for clinical treatment compared with the diagnosis of vitamin D deficiency. On one hand, we converted medians and inter-quartile ranges to means and standard deviations for vitamin D levels, with a non-Gaussian distribution, reducing the precision of a study (30). On the other hand, there was a lack of sufficient data extracted to identify the association between vitamin D levels and SGA.

An interesting finding in our subgroup analysis indicated that the sampling time point of maternal vitamin D was associated with risk of SGA. Maternal vitamin D deficiency was associated with 37% and 49% increased risk of SGA when maternal blood samples were measured before and after 16 wk of gestational age, respectively. These results are comparable with other meta-analyses. The pooled odds ratio was 1.77(95%CI:1.43,2.19) between maternal vitamin D deficiency and risk of SGA when the gestational age of sampling was <16 wk, and the pooled odds ratio was 2.69 (95%CI: 1.45,5.01) between maternal vitamin D deficiency and risk of SGA when the gestational age of sampling was >16 wk (20). There are three potential reasons for this inconsistency. First, Aghajafari’s review included pregnant women with HIV infection. Second, the review defined SGA as neonates with birth weights below the 5^th^ percentile for their gestational age. Third, the cut-off for vitamin D deficiency was below 37.5 nmol/L, whereas that of our study was below 50 nmol/L.

From the funnel plots in the current meta-analysis, there was no obvious publication bias. Moreover, there was moderate heterogeneity for the pooled OR between vitamin D deficiency and risk of SGA. Due to the association between vitamin D concentrations below 37.5nmol/L only being included in three studies and nearly nonexistent heterogeneity (*I*^2^=0%, *P*=0.94), we did not conduct sensitivity and subgroup analyses. For vitamin D concentrations below 50 nmol/L, our sensitivity analysis showed that in Netherlands (16) contributed to the heterogeneity. There may be other factors contributing to heterogeneity in the current meta-analysis. First, there is still a lack of clear and uniform standards for defining vitamin D deficiency. Second, the different assay methods used to quantify 25-OHD levels may be an important factor when evaluating the risk of vitamin D deficiency. Third, the seasons of sampling, pregnant women of race, pregnancy BMI, maternal age and smoking during pregnancy were confounding factors of the association between maternal vitamin D deficiency and risk of SGA.

Vitamin D has immunomodulatory roles during pregnancy that enable successful implantation by attenuating decidual T-cell function (33, 34). However, the biological mechanism through which vitamin D deficiency during pregnancy elevates the risk of SGA infants remains obscure. Increasing evidence demonstrates that vitamin D has an anti-inflammatory activity (35, 36). Additionally, maternal vitamin D deficiency may result in abnormal maternal calcium metabolism in the myometrium (37) or alter placental function (38, 39). Moreover, low maternal vitamin D may affect fetal bone in the case of low calcium intake (40) and lost regulation of glucose metabolism, which affects fetal mass (41).

Seasons and latitude were the influencing factors for vitamin D. Vitamin D deficiency is typical in high-latitude and during winters and no vacation in sunny regions (42, 43), characterized by short periods of sunlight exposure. The prevalence was 13.2% in Singapore (1 °N) and 40.1% in New South Wales (34 °S). Therefore, vitamin D supplementation is a feasible way for pregnant women to maintain sufficient vitamin D levels. However, the preventive effect of vitamin D supplementation on neonatal outcomes has not be recognized. For instance, a meta-analysis that included thirteen RCTs found that the incidence of GDM, preeclampsia, preterm birth and SGA were not influenced by vitamin D supplementation (44). Another meta-analysis included five RCTs showed a protective effect of vitamin D supplementation on low birthweight and a non-significant but suggestive effect on daily vitamin D supplementation on small for gestational age (RR=0.67,95%CI:0.40,1.11) (45). Therefore, additional larger randomized trials are required to define the benefits of vitamin D supplementation in reducing the incidence of SGA, and focusing on reducing SGA and its consequences are needed to more accurately evaluate the potential benefits of these low-cost interventions in the future. There were also several limitations in this meta-analysis. First, the effect of vitamin D levels under 50 nmol/L with risk of SGA should be observed. The effect of vitamin D deficiency with risk of SGA maybe overestimated when the SGA events induced by severe vitamin D deficiency (vitamin D concentration below 30 or 25 nmol/L) were combined into one group. Hence, it would be more accurate if the effects of the association between different cut-offs and risk of SGA were analyzed separately. Second, we were unable to show a dose-response relationship between the different levels of maternal vitamin D deficiency and risk of SGA. This may be due to the lack of sufficient data about different cut-offs of vitamin D deficiency and risk of SGA. This needs to be dealt with in future studies, and further work on what defines a standard normal and deficient level of 25-OHD in pregnancy is required. Third, the publication bias of our meta-analysis was only visualized through funnel plots but not quantification by Begg’s and Egger’s tests. Fourth, our meta-analysis was only based on observational studies, and the possibility of selection bias, misclassification bias related to exposure, and failure to consider residual or un-measured confounding factors cannot be ruled out. The assessment methods for vitamin D concentrations may also vary between the studies.

## Conclusion

Vitamin D deficiency was associated with an increased risk of small for gestational age. The pooled odds ratio was 1.41 (95%CI:1.14, 1.75) between the association of vitamin D levels less than 50 nmol/L and risk of SGA. For further studies, the dose-response effect of different cutoffs of vitamin D deficiency should be observed and researched. More studies are needed to better understand the effect of maternal vitamin D levels on risk of small for gestational age, and well-designed trials are required to determine the effect of vitamin D supplementation on the prevention of SGA in the future.

## Ethical considerations

Ethical issues (Including plagiarism, informed consent, misconduct, data fabrication and/or falsification, double publication and /or submission, redundancy, etc.) have been completely observed by the authors.

## References

[1] Holick MF (2007). Vitamin D deficiency. N Engl J Med, 357:266–281. 17634462 10.1056/NEJMra070553

[2] Bodnar LM Simhan HN Powers RW (2007). High prevalence of vitamin D insufficiency in black and white pregnant women residing in the northern United States and their neonates. J Nutr, 137:447–452. 17237325 10.1093/jn/137.2.447 PMC4288960

[3] Maghbooli Z Hossein-Nezhad A Karimi F (2008). Correlation between vitamin D3 deficiency and insulin resistance in pregnancy. Diabetes Metab Res Rev, 24:27–32. 17607661 10.1002/dmrr.737

[4] Zhang C Qiu C Hu FB (2008). Maternal plasma 25-hydroxyvitamin D concentrations and the risk for gestational diabetes mellitus. PLoS One, 3: e3753. 19015731 10.1371/journal.pone.0003753 PMC2582131

[5] Makgoba M Nelson SM Savvidou M (2011). First-trimester circulating 25-hydroxyvitamin D levels and development of gestational diabetes mellitus. Diabetes Care, 34:1091–1093. 21454797 10.2337/dc10-2264 PMC3114479

[6] Robinson CJ Wagner CL Hollis BW (2011). Maternal vitamin D and fetal growth in early-onset severe preeclampsia. Am J Obstet Gynecol, 204(6):556.e1–4. 21507371 10.1016/j.ajog.2011.03.022 PMC3136573

[7] Hensel KJ Randis TM Gelber SE (2011). Pregnancy-specific association of vitamin D deficiency and bacterial vaginosis. Am J Obstet Gynecol, 204(1):41.e1–9. 20887971 10.1016/j.ajog.2010.08.013

[8] Qin LL Lu FG Yang SH (2016). Does Maternal Vitamin D Deficiency Increase the Risk of Preterm Birth: A Meta-Analysis of Observational Studies. Nutrients, 8(5): E301. 27213444 10.3390/nu8050301 PMC4882713

[9] Merewood A Mehta SD Chen TC (2009). Association between vitamin D deficiency and primary cesarean section. J Clin Endocrinol Metab, 94(3):940–5. 19106272 10.1210/jc.2008-1217 PMC2681281

[10] O’Loan J Eyles DW Kesby J (2007). Vitamin D deficiency during various stages of pregnancy in the rat; its impact on development and behaviour in adult offspring. Psychoneuroendocrinology, 32:227–234. 17276604 10.1016/j.psyneuen.2006.12.006

[11] Strom M Halldorsson TI Hansen S (2014). Vitamin D measured in maternal serum and offspring neurodevelopmental outcomes: a prospective study with long-term follow-up. Ann Nutr Metab, 64:254–261. 25300268 10.1159/000365030

[12] Slancheva B Mumdzhiev H (2013). Small for gestational age newborns--definition, etiology and neonatal treatment. Akush Ginekol (Sofiia), 52:25–32. 23807977

[13] Gaudineau A (2013). Prevalence, risk factors, maternal and fetal morbidity and mortality of intrauterine growth restriction and small-for-gestational age. J Gynecol Obstet Biol Reprod (Paris), 42:895–910. 24216305 10.1016/j.jgyn.2013.09.013

[14] Hernandez MI Mericq V (2011). Metabolic syndrome in children born small-for-gestational age. Arq Bras Endocrinol Metabol, 55:583–589. 22218440 10.1590/s0004-27302011000800012

[15] Chen YH Fu L Hao JH (2015). Maternal vitamin D deficiency during pregnancy elevates the risks of small for gestational age and low birth weight infants in Chinese population. J Clin Endocrinol Metab, 100:1912–1919. 25774884 10.1210/jc.2014-4407

[16] Leffelaar ER Vrijkotte TG van Eijsden M (2010). Maternal early pregnancy vitamin D status in relation to fetal and neonatal growth: results of the multi-ethnic Amsterdam Born Children and their Development cohort. Br J Nutr, 104:108–117. 20193097 10.1017/S000711451000022X

[17] Zhou J Su L Liu M (2014). Associations between 25-hydroxyvitamin D levels and pregnancy outcomes: a prospective observational study in southern China. Eur J Clin Nutr, 68:925–930. 24865483 10.1038/ejcn.2014.99

[18] Morgan C Dodds L Langille DB (2016). Cord blood vitamin D status and neonatal outcomes in a birth cohort in Quebec, Canada. Arch Gynecol Obstet, 293:731–738. 26404451 10.1007/s00404-015-3899-3 PMC5023425

[19] Rodriguez A Garcia-Esteban R Basterretxea M (2015). Associations of maternal circulating 25-hydroxyvitamin D3 concentration with pregnancy and birth outcomes. BJOG, 122:1695–1704. 25208685 10.1111/1471-0528.13074

[20] Aghajafari F Nagulesapillai T Ronksley PE (2013). Association between maternal serum 25-hydroxyvitamin D level and pregnancy and neonatal outcomes: systematic review and meta-analysis of observational studies. BMJ, 346:f1169. 23533188 10.1136/bmj.f1169

[21] von Elm E Altman DG Egger M (2014). The Strengthening the Reporting of Observational Studies in Epidemiology (STROBE) Statement: guidelines for reporting observational studies. Int J Surg, 12:1495–1499. 25046131 10.1016/j.ijsu.2014.07.013

[22] Hozo SP Djulbegovic B Hozo I (2005). Estimating the mean and variance from the median, range, and the size of a sample. BMC Med Res Methodol, 5:13. 15840177 10.1186/1471-2288-5-13 PMC1097734

[23] Bodnar LM Catov JM Zmuda JM (2010). Maternal serum 25-hydroxyvitamin D concentrations are associated with small-for-gestational age births in white women. J Nutr, 140:999–1006. 20200114 10.3945/jn.109.119636 PMC2855265

[24] Burris HH Rifas-Shiman SL Camargo CA Jr (2012). Plasma 25-hydroxyvitamin D during pregnancy and small-for-gestational age in black and white infants. Ann Epidemiol, 22:581–586. 22658824 10.1016/j.annepidem.2012.04.015 PMC3396717

[25] Boyle VT Thorstensen EB Mourath D (2016). The relationship between 25-hydroxyvitamin D concentration in early pregnancy and pregnancy outcomes in a large, prospective cohort. Br J Nutr, 116:1409–1415. 27753425 10.1017/S0007114516003202

[26] Fernandez-Alonso AM Dionis-Sanchez EC Chedraui P (2012). First-trimester maternal serum 25-hydroxyvitamin D_3_ status and pregnancy outcome. Int J Gynaecol Obstet, 116:6–9. 21959069 10.1016/j.ijgo.2011.07.029

[27] Gernand AD Simhan HN Klebanoff MA (2013). Maternal serum 25-hydroxyvitamin D and measures of newborn and placental weight in a U.S. multicenter cohort study. J Clin Endocrinol Metab, 98:398–404. 23162094 10.1210/jc.2012-3275 PMC3537090

[28] Gernand AD Simhan HN Caritis S (2014). Maternal vitamin D status and small-for-gestational-age offspring in women at high risk for preeclampsia. Obstet Gynecol, 123:40–48. 24463662 10.1097/AOG.0000000000000049 PMC3914014

[29] Ong YL Quah PL Tint MT (2016). The association of maternal vitamin D status with infant birth outcomes, postnatal growth and adiposity in the first 2 years of life in a multi-ethnic Asian population: the Growing Up in Singapore Towards healthy Outcomes (GUSTO) cohort study. Br J Nutr, 116:621–631. 27339329 10.1017/S0007114516000623 PMC4967353

[30] Schneuer FJ Roberts CL Guilbert C (2014). Effects of maternal serum 25-hydroxyvitamin D concentrations in the first trimester on subsequent pregnancy outcomes in an Australian population. Am J Clin Nutr, 99:287–295. 24257720 10.3945/ajcn.113.065672

[31] Weinert LS Reichelt AJ Schmitt LR (2016). Vitamin D Deficiency Increases the Risk of Adverse Neonatal Outcomes in Gestational Diabetes. PLoS One, 11:e0164999. 27764194 10.1371/journal.pone.0164999 PMC5072629

[32] Wei SQ Qi HP Luo ZC (2013). Maternal vitamin D status and adverse pregnancy outcomes: a systematic review and meta-analysis. J Matern Fetal Neonatal Med, 26:889–899. 23311886 10.3109/14767058.2013.765849

[33] Liu NQ Hewison M (2012). Vitamin D, the placenta and pregnancy. Arch Biochem Biophys, 523:37–47. 22155151 10.1016/j.abb.2011.11.018

[34] Vieth R (2007). Vitamin D toxicity, policy, and science. J Bone Miner Res, 22 Suppl 2:V64–68. 18290725 10.1359/jbmr.07s221

[35] Luderer HF Nazarian RM Zhu ED (2013). Ligand-dependent actions of the vitamin D receptor are required for activation of TGF-beta signaling during the inflammatory response to cutaneous injury. Endocrinology, 154:16–24. 23132743 10.1210/en.2012-1579 PMC3529380

[36] Cotechini T Komisarenko M Sperou A (2014). Inflammation in rat pregnancy inhibits spiral artery remodeling leading to fetal growth restriction and features of preeclampsia. J Exp Med, 211:165–179. 24395887 10.1084/jem.20130295 PMC3892976

[37] Buxton IL (2004). Regulation of uterine function: a biochemical conundrum in the regulation of smooth muscle relaxation. Mol Pharmacol, 65:1051–1059. 15102932 10.1124/mol.65.5.1051

[38] Evans KN Bulmer JN Kilby MD (2004). Vitamin D and placental-decidual function. J Soc Gynecol Investig, 11:263–71. 10.1016/j.jsgi.2004.02.002 15219879

[39] Liu NQ Kaplan AT Lagishetty V (2011). Vitamin D and the regulation of placental inflammation. J Immunol, 186:5968–5974. 21482732 10.4049/jimmunol.1003332

[40] Galthen-Sorensen M Andersen LB Sperling L (2014). Maternal 25-hydroxyvitamin D level and fetal bone growth assessed by ultrasound: a systematic review. Ultrasound Obstet Gynecol, 44:633–640. 24891235 10.1002/uog.13431

[41] Yap C Cheung NW Gunton JE (2014). Vitamin D supplementation and the effects on glucose metabolism during pregnancy: a randomized controlled trial. Diabetes Care, 37:1837–1844. 24760259 10.2337/dc14-0155

[42] Deplanque X Wullens A Norberciak L (2017). Prevalence and risk factors of vitamin D deficiency in healthy adults aged 18–65 yr in northern France. Rev Med Interne, 38(6):368–373. 28161111 10.1016/j.revmed.2016.12.013

[43] Choi R Kim S Yoo H (2015). High prevalence of vitamin D deficiency in pregnant Korean women: the first trimester and the winter season as risk factors for vitamin D deficiency. Nutrients, 7:3427–3448. 25970148 10.3390/nu7053427 PMC4446760

[44] Perez-Lopez FR Pasupuleti V Mezones-Holguin E (2015). Effect of vitamin D supplementation during pregnancy on maternal and neonatal outcomes: a systematic review and meta-analysis of randomized controlled trials. Fertil Steril, 103:1278–88.e4. 25813278 10.1016/j.fertnstert.2015.02.019

[45] Thorne-Lyman A Fawzi WW (2012). Vitamin D during pregnancy and maternal, neonatal and infant health outcomes: a systematic review and meta-analysis. Paediatr Perinat Epidemiol, 26 Suppl 1:75–90. 22742603 10.1111/j.1365-3016.2012.01283.x PMC3843348

